# The Characteristics and Survival Potential Under Sub-lethal Stress of Mesenchymal Stromal/Stem Cells Isolated from the Human Vascular Wall

**DOI:** 10.1093/stmcls/sxac066

**Published:** 2022-09-13

**Authors:** Carmen Ciavarella, Sabrina Valente, Gianandrea Pasquinelli

**Affiliations:** DIMES - Department of Experimental, Diagnostic and Specialty Medicine, University of Bologna, Bologna, Italy; DIMES - Department of Experimental, Diagnostic and Specialty Medicine, University of Bologna, Bologna, Italy; DIMES - Department of Experimental, Diagnostic and Specialty Medicine, University of Bologna, Bologna, Italy; Subcellular Nephro-Vascular Diagnostic Program, Pathology Unit, IRCCS Azienda Ospedaliero-Universitaria di Bologna, Bologna, Italy

**Keywords:** mesenchymal stromal/stem cells, human vascular wall, sub-lethal stress, survival

## Abstract

Mesenchymal stromal/stem cells (MSCs) have been identified in multiple human tissues, including the vascular wall. High proliferative potential, multilineage, and immunomodulatory properties make vascular MSCs promising candidates for regenerative medicine. Indeed, their location is strategic for controlling vascular and extra-vascular tissue homeostasis. However, the clinical application of MSCs, and in particular vascular MSCs, is still challenging. Current studies are focused on developing strategies to improve MSC therapeutic applications, like priming MSCs with stress conditions (hypoxia, nutrient deprivation) to achieve a higher therapeutic potential. The goal of the present study is to review the main findings regarding the MSCs isolated from the human vascular wall. Further, the main priming strategies tested on MSCs from different sources are reported, together with the experience on vascular MSCs isolated from healthy cryopreserved and pathological arteries. Stress induction can be a priming approach able to improve MSC effectiveness through several mechanisms that are discussed in this review. Nevertheless, these issues have not been completely explored in vascular MSCs and potential side effects need to be investigated.

## Graphical Abstract

**Figure:**
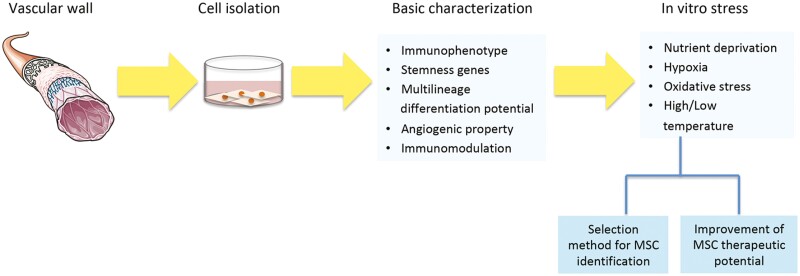
The vascular wall represents a reservoir of mesenchymal stromal/stem cells (MSCs). The exposure to stressful conditions in vitro can improve MSC therapeutic performance, for successful clinical use. These strategies can be applied to select and identify MSCs. This figure was created adapting Servier Medical Art templates (licensed under Creative Commons Attribution 3.0 Unported License smart.servier.com).

**Significance Statement**

Vascular wall represents a reservoir of progenitors cells, including mesenchymal stromal/stem cells (MSCs) endowed of high proliferative, regenerative, and immunomodulatory potential. Thus, the improvement of MSC collection and characterization from human vascular wall should be encouraged in the view of standardized procedures. Moreover, the search for priming approaches addressed at enhanching vascular MSC applications is expected to be a promising strategy in the clinical field. This review highlights how stress can shape the features and functionality of MSCs, through approaches that can be extended to vascular MSCs.

## Mesenchymal Stromal/Stem Cells: Properties, Origin, and Applications

Mesenchymal stromal/stem cells (MSCs) are adult stem cells, endowed with self-renewal and multipotency capabilities. According to the International Society for Cellular Therapy, the minimum criteria for MSC identification are as follows: (1) adherence to plastic; (2) expression of a specific subset of cell surface markers (ie, CD90, CD105, CD73), lack of CD14, CD45, CD34, and human leukocyte antigen-DR (HLA-DR); (3) in vitro differentiation into the adipogenic, osteogenic, and chondrogenic lineage.^1,2^ Niches of MSCs have been identified in several human tissues, including bone-marrow,^3^ peripheral blood,^4^ adipose tissue,^5,6^ and vascular wall.^7-9^ MSCs can control tissue homeostasis, renewal and damage by homing to the damaged tissues and replacing dead cells. Further, MSCs can regulate the innate and adaptive immune response by suppressing the inflammatory process.^10^ MSCs possess an undisguised and promising regenerative potential, even though their efficacy for the therapy of human disorders is still challenging and under debate. The lack of standardized protocols for cell isolation, the cell heterogeneity, and the poor cell survival rate after transplantation represent serious limitations. Therefore, the search for methods of enhancing MSC properties and their therapeutic potential is under investigation.

The origin of the tissue also influences the MSC potential; indeed, MSCs isolated from distinct tissues display different biological features, including the immunomodulatory property. In this regard, the vascular wall is an important reservoir of MSCs, which critically regulate many biological processes, involved both in normal and pathological settings of vascular and extravascular tissues.^11^ This concern is crucial for the safe clinical application of MSCs, like in intravascular infusion. Indeed, perivascular MSCs from different tissues express differential levels of highly procoagulant tissue factor TF/CD142, thus influencing the hemocompatibility and the safety of the cell engraftment.^12,13^

The main goal of the present review is to illustrate the sources and the characteristics of vascular MSCs, focusing on the resistance to stress in vitro and ex vivo. This feature can be considered a marker and a selection criterion for MSCs. Further, the exposure to sub-lethal cell stressors might be useful as in vitro strategy to empower MSC therapeutic potential.

## Types and Characteristics of Vascular Progenitors

The vascular wall hosts a niche of stem/progenitor cells, including MSCs, that perform a pivotal role in keeping vascular homeostasis and regeneration. This niche is heterogeneous and comprises cell populations of different sources: bone marrow, circulation, large and small blood vessels.^11,14^ Bone marrow-derived vascular progenitors include endothelial progenitor cells (EPCs), able to differentiate into mature endothelial cells (ECs) and give rise to new blood vessels in vivo.

In the human vascular wall of adult blood vessels, the vasculogenic zone has been described as a border zone between the media and the adventitia layer. In this specific zone, a different subpopulation of progenitor cells, including EPC and stem cells with the ability to differentiate into mature endothelial cells, hematopoietic and local macrophages, are located; further, other cell populations such as hematopoietic as well as mesenchymal stem cells may reside.^15^ From these arterial segments, the resident vascular multipotent stem cells were successfully isolated showing stem properties comparable to those typical of the bone marrow MSC and with marked ability to differentiate in endothelial cells^7^ and multilineage mesodermal potential.^8^ The tunica adventitia is extensively studied for the presence of several kinds of perivascular mesenchymal progenitors, including osteoprogenitor CD10^+^, CD107a^low^, and CD140a^+^ cells who participate in bone formation and repair, as reviewed in Xu et al.^16^ This topic will be discussed in the specific section.

## Vascular Progenitors in Small Blood Vessels

Regarding the small blood vessels, the presence of pericytes was firstly observed by Rouget in 1873.^17^ Pericytes are connected with endothelial cells (ECs) of capillaries and microvessels through multiple junctions, sharing the basal membrane.^18^ Pericytes are important regulators of many vascular processes, such as vascular development, tone, stabilization, permeability, and pressure in microcirculation.^19^ Further, pericytes participate in the angiogenic process by triggering EC differentiation and allowing the vascular network maturation.^20^ Pericytes share with MSCs many properties, like the differentiation potential into the adipogenic, chondrogenic, osteogenic, and myogenic lineage.^21^ However, the discrimination between pericytes and MSCs has not been established yet. Under normal tissue homeostasis, pericytes dynamically express molecules such as platelet-derived growth factor receptor beta (PDGFR-β), CD13 (alanine aminopeptidase), cluster of differentiation 146 (CD146), a molecule that is also expressed by endothelial cells, alpha-smooth muscle actin (α-SMA, also shared with smooth muscle cells and other perivascular cytotypes). On the contrary, the perivascular MSCs in normal conditions are visible outside the capillary wall where they appear free in the extracellular matrix without a basement membrane. They express the typical MSC marker signature and are endowed with differentiation capacity in common mesengenic lines. A recent study proposed the expression of early B-cell factor 1 (EBF-1), a gene involved in B-cell differentiation during tissue development,^22^ as a possible tool to identify and distinguish pericytes from MSCs.^23^ Even though pericytes contribute to tissue repair and regeneration, many disease conditions can affect their nature and compromise the healing property. Indeed, under a pathological context, pericytes undergo pericyte-fibroblast transition that has been demonstrated having repercussions both in the instability and leakage of tumor angiogenesis and in the alteration of crosstalk with tumor/stromal cells in tumor microenvironment.^24^

To make the issue even more complex, there is the possibility that further perivascular cytotypes exist; at this regard, telocytes are described as slender and extremely thin bipolar CD34 + cells (a marker shared with endothelial cells).^25^ Telocytes are present in all tissues in a perivascular location with functions of communication between distinct sites and tensile support to solid structures in contact with the extracellular matrix. Further, in a pathological vascular context, for example in atherosclerosis, myofibroblasts are described as transitional cells between fibroblasts and smooth muscle cells; these cells have always been described in pathological/reparative contexts.^26^

## MSCs Resident in Human Healthy and Diseased Blood Vessels

A wide range of evidence has highlighted the presence of MSC niches within the wall of adult blood vessels, involved in the regulation of several biological processes from tissue homeostasis and neovascularization, to disease progression (ie, extracellular matrix degradation, vascular remodeling).

A population of CD44^+^CD90^+^CD73^+^CD34^−^CD45^−^ multipotent cells was obtained from human internal thoracic arteries through mechanic/enzymatic digestion.^27^ Further, these cells contributed to the vessel morphogenesis in vivo, when co-implanted with human umbilical vein endothelial cells (HUVEC).^27^

Campagnolo and colleagues isolated a population of progenitor cells negative to CD31 and von Willebrand factor, positive to CD34, with proangiogenic potential and able to induce neovascularization in a mouse model of hind limb ischemia.^28^ In this study, cells were isolated from the veins through enzymatic digestion followed by selection methods, including immunomagnetic beads and fluorescence cell sorting. Yang and colleagues compared the MSCs isolated from artery, vein and small vessels in the adipose tissue, with adipose-derived stem/stromal cells (ASCs).^29^ They showed that vascular cells were comparable for basic mesenchymal characteristics, including morphology and immunophenotype, but differed from each other as regards the differentiation properties. Compared to ASCs, artery-derived progenitors were more effective at calcium deposition, whereas vein and small vessel ones resulted more prone to network formation.^29^

MSCs characterized by multilineage differentiation potential, immunomodulation, and clonogenic capacity were isolated from fresh and cryopreserved carotid artery, aortic arch, thoracic and abdominal aorta, and femoral artery, through enzymatic digestion.^8,30^ An intriguing observation emerged from the identification of MSCs in cryopreserved healthy arteries of multi-tissue donors and pathological arteries, suggesting the capacity of vascular progenitors to counteract multiple kinds of stress and survive. Details will be discussed in the next section.

MSCs were also identified and obtained from pathological arteries belonging to patients affected by abdominal aortic aneurysm (AAA, AAA-MSCs), and interestingly these cells exhibited compromised features like poor immunosuppressive activity and abnormal osteogenic differentiation.^31,32^ The successful isolation of MSCs from pathological vascular biopsies supported the resistance of vascular MSCs to the extracellular microenvironment adverse to cell growth and health: inflammation, hypoxia, and oxidative stress are some of the most common pathogenic processes involved in AAA disease.

More recently, Michelis et al identified a CD90+ cell population within healthy and diseases arteries, and provided a transcriptomic signatures associated with the atherosclerosis pathogenesis.^33^ Here, tissues were processed through enzymatic digestion followed by cell sorting through flow cytometry.

A comparison performed between human saphenous vein (SV-MSCs) and bone marrow MSCs (BM-MSCs) elucidated that these 2 cell populations share phenotype, gene signature and differentiation potential, whereas the SV-MSCs resulted more responsive to inflammatory stimulation by exerting a higher immunosuppressive activity on human peripheral blood mononuclear cells (PBMCs) proliferation in vitro.^34^

## MSCs and Stress: Major Counteracting Strategies

Stress is a condition elicited by a series of harmful stimuli that seriously affects cell healthy, with potentially detrimental consequences. A toxic environment, such as occurs under nutrient deprivation, oxidative stress, hypoxia, heat stress, inflammation, and metabolic alterations, activates specific signaling pathways resulting in cell repair or alternatively in cell senescence and apoptosis. However, the role of stress in MSC biology is controversial. Indeed, stress shapes the fate and the functions of MSCs, which undergo a specific lineage differentiation to repair the damaged tissue and replace its function. In this view, stress is an important trigger of MSC activation.

In general, stem cells can respond to stress by entering into a quiescent state, that is a reversible cell cycle arrest in G0/G1 phase.^35,36^ During this phase, cells undergo a metabolic slowing, inhibition of transcription and translation processes, and differential gene expression signature. Indeed, it has been observed a downregulation of genes involved in DNA replication and cell proliferation (including cyclin A2, cyclin B1, cyclin E2, survivin), and an upregulation of cell-fate regulating genes like FOXO3.^35^ FOXO3 belongs to the family of the forkhead transcription factors, involved in cell proliferation, survival, and metabolic pathways,^37^ and it stimulates skeletal muscle stem cells (SCs) entry in quiescence in adult muscle regeneration.^38^

Several conditions, such as cell-cell interactions, microenvironment factors, and molecular signaling, induce and regulate MSC quiescence. In vitro serum deprivation takes MSCs under a non-proliferative state with low RNA and protein synthesis, which can be reversed to active proliferation by serum and nutrient reintroduction.^39^ Another defence mechanism is autophagy, a program addressed at preserving the metabolic and bioenergetic functions of the cell. Autophagy is mainly induced by nutrient deprivation, DNA damage, endoplasmic reticulum stress, hypoxia, and oxidative stress.

Accumulating evidence shows that MSCs are highly resistant under a stressful environment; indeed, it has been suggested that preconditioning MSCs with hypoxic or oxidative stress stimuli may potentiate their regenerative and reparative features. Indeed, such conditions implicate a huge metabolic stress for MSCs, and a consequent adaptation to the adverse environment for survival purposes. The quiescence preconditioning improved MSC viability under low oxygen conditions or glucose depletion.^39^ Quiescence induced by serum starvation in human endometrial MSCs (eMSCs) exerted a protective effect from sub lethal heat shock.^40^ Further, heat stress was able to promote multilineage differentiation in human bone-marrow MSCs, in particular in the myogenic^41^ and in the osteogenic lineages.^42^ A study performed on rat bone-marrow MSCs showed that exposure to low doses of H_2_O_2_ improved cell survival and potency.^43^ Similarly, preconditioning human adipose-derived MSCs (haASCs) after cryopreservation with low concentration of H_2_O_2_ promoted adhesion, migration, and resistance to oxidative stress.^44^ Long- and short-term hypoxia improved porcine and human MSCs functions, such as proliferation, self-renewal, immunomodulatory property, and metabolic activity.^45^ Nutrient-deprivation inhibits the mammalian target of rapamycin complex-1 (mTORC1), resulting in cell metabolic arrest and autophagy induction.^46^ In this regard, intriguing studies support the role of fasting in delaying the aging process, as demonstrated in rodent models where intermittent or periodic fasting was associated with a lower incidence of diseases such as cancers and neurodegeneration.^47^ The main strategies that MSCs adopt to counteract stress are summarized in Table 1.

**Table 1.** The potential of MSCs to counteract stress.

Type of stress	Model	Response
Nutrient deprivation	Human bone marrow MSCs	Quiescence and improved viability under low oxygen and glucose levels, autophagy^23^
Heat stress	Human endometrial MSCs Human bone marrow MSCs	Higher tolerance in quiescent MSCs^24^ Promote myoblast^25^ and osteogenic^26^ differentiation
Oxidative stress	Rat bone marrow MSCs Human adipose-derived MSCs	Increased survival and potency under non-lethal doses of H_2_O_2_^27^ Higher resistance to oxidative stress^28^
Hypoxia	Human and porcine bone marrow MSCs	Improved survival and therapeutic properties ^29^

A list of the main strategies adopted by MSCs from animal and human adult tissues when exposed to different kinds of stress (nutrient deprivation, heat stress, oxidative stress, hypoxia). These mechanisms can be exploited in vitro to strengthen the therapeutic properties of MSCs for clinical applications.

Abbreviation: MSC, mesenchymal stem cells.

The recent discovery of micro-RNA (miRNA) involvement in stem cell differentiation, pluripotency, and proliferation has proposed their role in the maintenance and modulation of MSC quiescence. miRNA are small non coding RNAs, able to regulate target gene expression by degrading mRNA or inhibition the translation process. It has been shown that the ablation of miRNA processing enzyme Dicer interrupts quiescence, inducing premature apoptosis of muscular stem cells (MuSCs).^48^ In addition, miR-489 stimulates the entry in quiescence in MuSCs.^48^ A study performed on bone marrow MSCs, elucidated the downregulation of miR-206 at mitigating senescence and dysfunction caused by H_2_O_2_ exposure.^49^

In addition to preconditioning, the manipulation of genes regulating cell growth and survival pathways has also been proposed to achieve MSCs with higher therapeutic performance.^50^

An important consideration about the safety of the above-mentioned strategies to enrich MSC therapeutic features should be remarked, especially in regard to the tumor growth. Indeed, the quiescence/dormancy induction can exert a dual role, because in addition to the enhancement of therapeutic properties, also can select cells that can resist to cancer therapy. Dormant/quiescent cancer cells are mainly concentrated in G0 phase, and are known as cancer stem cells (CSCs) for their self-renewal and differentiation abilities.^51^ This cell component can survive cancer treatment, ie, chemo or radiotherapy, becoming a source of residual cancer cells that can re-activate after therapy and promote tumor relapse.^52^

## Vascular MSCs Ability to Survive Under Sub-lethal Stress In Vitro

The induction of sub-lethal stress in vitro may represent a strategy to characterize and select MSCs with ancestral stemness features. According to the standard culture conditions, cells are kept in a controlled atmosphere with 37 °C, 21% O_2_ and 5% CO_2_. However, it is well known that stem cells reside in a niche, which is a protected hypoxic microenvironment that preserves stem cell self-renewal. Thus, it can be hypothesized that low oxygen culture can mimic the niche microenvironment and potentiates stemness properties, including the resistance to stress.

**Figure 1.**
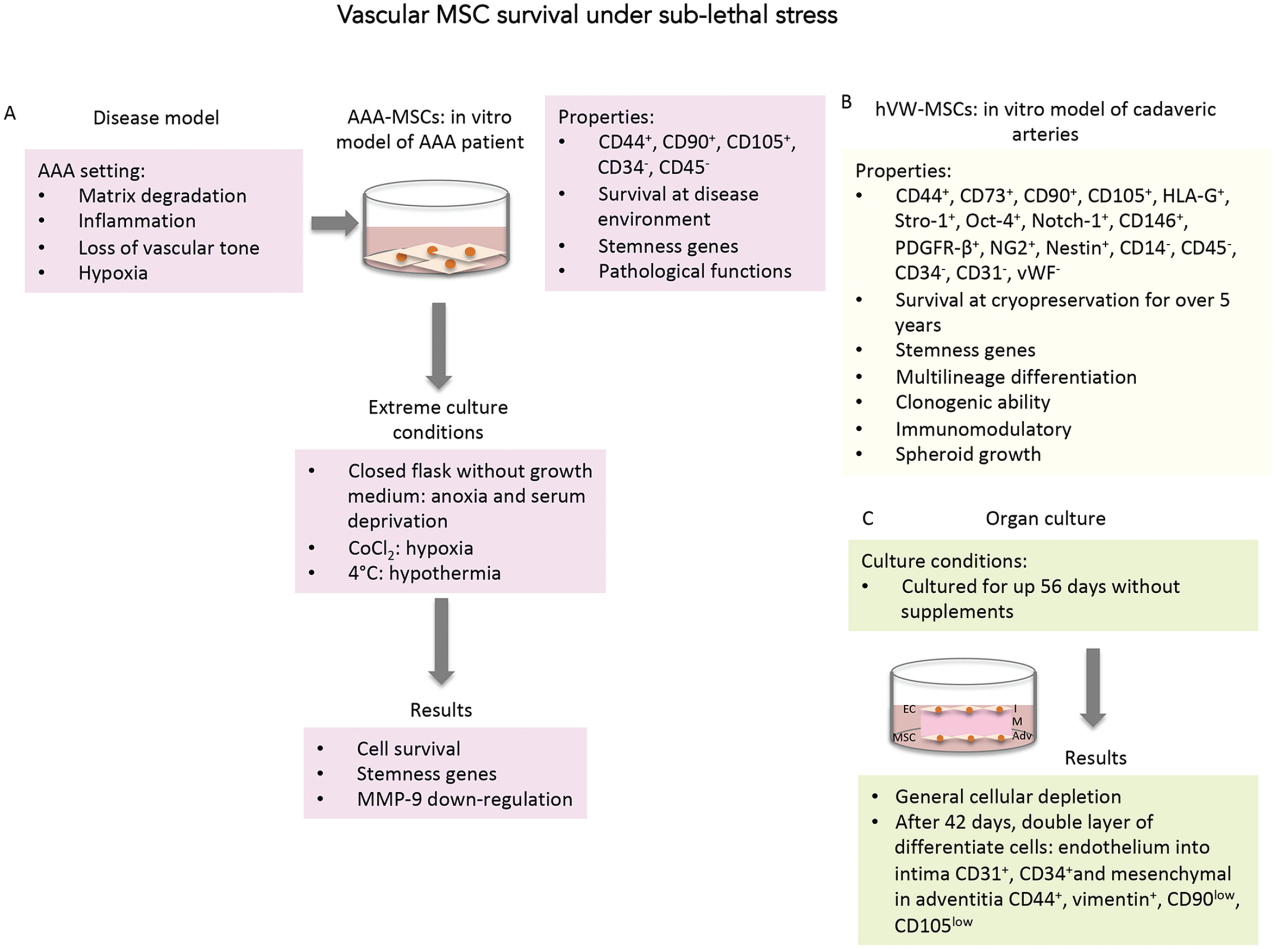
Features of vascular MSCs isolated from pathological and healthy arteries. Summary of the characteristics of vascular MSCs isolated both from disease and healthy context. (**A**) Characterization and features of MSCs isolated from disease setting and exposed to sub-lethal stress induced by: nutrient and oxygen deprivation, and low temperature. (**B**) Characterization and features of MSCs isolated from human post-mortem arteries cryopreserved over than 5 years in liquid nitrogen. (**C**) Characterization of long-term organ culture performed on human fresh femoral arteries. Abbreviations: AAA-MSCs, abdominal aortic aneurysm-mesenchymal stem cells; hVW-MSCs, human vascular wall-mesenchymal stromal/stem cells; CoCl_2_, cobalt chloride; EC, endothelial cells; I, intima tunica; M, media tunica; Adv, adventitia tunica.

Our research group provided the evidence that vascular MSCs isolated from human abdominal aortic aneurysm (AAA), can survive extreme culture conditions (Fig. 1A). Then, we tested AAA-MSCs behavior and survival under extreme culture conditions, such as nutrient deprivation, low oxygen, and hypothermia.^53^ The culture with glucose-free medium in a closed flask to reduce oxygen passage, the administration of cobalt chloride (CoCl_2_) as a chemical inducer of hypoxia, and the culture for 1 week at 4 °C to mimic hypothermia were shown to select a viable cell population. Indeed, after the first observation of drastic changes like altered cell morphology and reduced cell number, a resistant cell cluster grew up and reached confluence. The molecular analysis highlighted the maintenance of the stemness profile through the expression of self-renewal genes OCT-4, NANOG, SOX-2. Further, AAA-MSCs after being exposed to stress displayed a significant downregulation of the MMP-9 gene, encoding for a proteolytic enzyme involved in extracellular matrix degradation (ECM) and highly expressed in AAA tissues. This result suggested that such extreme culture conditions can reverse vascular MSC phenotype, and that MMP-9 could control MSC fate and differentiation, by regulating matrix-cell interactions that are crucial to MSC biology.^54^ Further investigations to confirm the role of MMP-9 in vascular MSC fate and whether sub-lethal stress can enrich the MSC stemness profile need to be explored.

## Vascular MSCs Survival from Post-mortem Tissues

Human vascular segments, collected from post-mortem donors and unused in vascular surgery, could be an alternative and inexhaustible source of multipotent mesenchymal stromal/stem cells for potential applications in regenerative medicine and, gene and MSC-based therapies.

In that regard, our research group reported the isolation of a cadaveric cell population with MSC characteristics and properties from human cadaveric arteries of young and healthy multi-tissue donors (nonheart beating) at 4 days post-mortem and stored in liquid nitrogen in tissue-banking facilities for more than 5 years (Fig. 1B). The human cadaveric-MSCs (hC-MSCs) were in vitro expanded for at least 14 passages, exhibiting high proliferation rate and clonogenic property. The immunophenotype revealed the positivity to mesenchymal (CD44, CD73, CD90, CD105, HLA-G), stemness (Stro-1, Oct-4, Notch-1), pericyte (CD146, PDGFR-β, NG2) and neuronal (Nestin) markers, while the hematopoietic and vascular ones were absent. Furthermore, hC-MSCs showed a differentiating capacity in multiple mesodermal lineages after exposure to specific induction media, an attitude to form floating spheroids when grown in suspension and a propensity to modulate the immune response when cocultured in the presence of activated peripheral blood mononuclear cells. These results suggest that in cadaveric vascular tissues persist MSCs capable of surviving to ischemic damage, absence of oxygen and nutrients, and freezing and thawing damages.^30^

In another previous study, we have described a spontaneous vascular wall remodeling occurring in a long-term organ culture (Fig. 1C). Fragments of fresh adult human femoral arteries collected from heart-beating multiorgan donors were kept in culture for up 56 days in absence of growth factors and blood flow. A complete depletion of all vascular cytotypes occurred during organ culture, conversely an opposite trend was observed from the 42th day until the end of the organ culture. Of interest, both intimal and adventitial sides were lined by a continuous layer of spindle cells properly differentiated into endothelium (CD31^+^ and CD34+) in intima and in mesenchymal lineage (CD44+, vimentin^+^, CD90^low^, and CD105^low^) in adventitia.^55^

## Ethical Considerations

The use of MSCs obtained from adult tissues gives the chance to investigate MSC potential for therapeutic applications, overcoming the ethical concerns and controversies posed by research on embryonic stem cells. In addition to adult tissues, it has been recently demonstrated the isolation and capabilities of MSCs from human cadavers, other than the vascular wall. The main research on this topic has been extensively reviewed in Cieśla and Tomsia.^56^ As seen from our experience on post-mortem vascular tissues, MSCs from cadaveric tissues retain stemness and differentiation features. Thus, the utilization of cadaveric sources for the isolation of MSCs provides for a promising investigation field to allow MSC-based therapies in terms of safety and effectiveness. However, also for cadaveric tissues, legal and ethical limitations have been recently pointed out. This issue opens novel challenges in the MSC research field.

## Conclusions

The vascular wall constitutes a reservoir of progenitor cells, particularly MSCs with a unique reparative and regenerative potential. The existence of this vasculogenic niche is intriguing, due to its strategic location. Indeed vascular wall MSCs can regulate the homeostasis and the function of extra-vascular tissues, contributing to tissue regeneration and repair in presence of injury. Many technical and biological issues, first of all, the lack of a standardized protocol for isolating and expanding MSCs, and, as recently outlined, the hemocompatibility, are objective limitations to their clinical use. In addition, the plasticity of vascular MSCs makes them susceptible to microenvironment changes, which determine a pathological switch with potentially detrimental effects. The strategies able to control the stress-activated signaling pathways, like those induced by hypoxia or nutrient deprivation, are promising and need to be investigated and validated. This approach could be useful for MSC isolation/identification, and for the enhancement of MSC features for clinical purposes, paying attention to the safety of the candidate method and taking into consideration of the potential tumorigenic risk.

## Data Availability

No new data were generated or analyzed in support of this research.
